# Mobile phone intervention for increasing adherence to treatment for type 2 diabetes in an urban area of Bangladesh: protocol for a randomized controlled trial

**DOI:** 10.1186/s12913-014-0586-1

**Published:** 2014-11-26

**Authors:** Sheikh Mohammed Shariful Islam, Andreas Lechner, Uta Ferrari, Guenter Froeschl, Dewan Shamsul Alam, Rolf Holle, Jochen Seissler, Louis W Niessen

**Affiliations:** Center for Control of Chronic Diseases, International Center for Diarrhoeal Diseases Research, Bangladesh (ICDDR,B), 68, Shaheed Tajuddin Ahmed Sarani, Mohakhali, Dhaka 1212 Bangladesh; Center for International Health (CIH), Ludwig-Maximilians-Universität (LMU), Leopoldstrasse 7, 80802 Munich, Germany; Diabetes Center, Diabetes Research Group, Medizinische Klinik und Poliklinik IV, Ludwig-Maximilians-Universität, Ziemssenstr. 1, 80336 Munich, Germany; Economic Evaluations, Helmholtz Zentrum München (GmbH), German Research Center for Environmental Health, Munich, Germany; Centre for Applied Health Research and Delivery, Liverpool School of Tropical Medicine, Pembroke Place, Liverpool, L3 5QA UK

## Abstract

**Background:**

Mobile phone technologies including SMS (short message service) have been used to improve the delivery of health services in many countries. However, data on the effects of mobile health technology on patient outcomes in resource-limited settings are limited. The aim of this study therefore is to measure the impact of a mobile phone SMS service on treatment success of newly diagnosed type 2 diabetes in an urban area of Bangladesh.

**Methods/design:**

This is a single-centred randomized controlled intervention trial (prospective) comparing standard-of-care with standard-of-care plus a mobile phone-based SMS intervention for 6 months. A total of 216 participants with newly diagnosed type 2 diabetes will be recruited. Data will be collected at the outpatient department of Bangladesh Institute of Health Science (BIHS) hospital at baseline and after 6 months. The primary outcome measure will be change in HbA1c between baseline and 6 months. The secondary outcome measures are self-reported medication adherence, clinic attendance, self-reported adoption of healthy behaviours, diabetes knowledge, quality of life and cost effectiveness of the SMS intervention. The inclusion criteria will be as follows: diagnosed as patients with type 2 diabetes by the BIHS physician, using oral medication therapy, living in Dhaka city, registered with the BIHS hospital, using a mobile phone, willing to return for follow up after 6 months and providing written informed consent. Participants will be allocated to control and intervention arms after recruitment using a randomization software. Data will be collected on socio-demographic and economic information, mobile phone use and habits, knowledge of prevention, management and complications of diabetes, self-perceived quality of life assessment, self-reported diseases, medical history, family history of diseases, medication history, medication adherence, health seeking behaviour, tobacco use, physical activity, diet, mental health status, life events and disability, anthropometric measurements of weight, height, blood pressure and blood tests for HbA1c.

**Discussion:**

Mobile phone SMS services have the potential to communicate with diabetes patients and to build awareness about the disease, improve self-management and avoid complications also in resource-limited setting. If this intervention proves to be efficient and cost-effective in the current trial, large-scale implementation could be undertaken.

**Trial registration:**

DRKS00005188.

## Background

Diabetes mellitus, particularly type 2, is one of the most significant public health challenges of the 21^st^ century. More than 80% of diabetes related deaths occur in developing countries [1]. It is estimated that about 8.4 million people living in Bangladesh are suffering from diabetes already and by 2030, that number is expected to double, placing Bangladesh among the top five countries in terms of the absolute number of people living with diabetes [2]. Population data support an increasing trend in diabetes prevalence in Bangladesh, especially in urban areas where the prevalence is 8.1% compared to 2.3% in rural areas [3].

Non-adherence rates for therapies of chronic illness and for lifestyle changes are high with estimates ranging from 36% to 93% and averaging only 50% in developed countries [4,5]. Lack of adherence often leads to increased use of healthcare services, poor quality of life and increased healthcare costs [6,7]. In Bangladesh, adherence to medication is low due to several complex issues along with poor clinic attendance. This often leads to insufficient glycaemic control and avoidable life threatening complications. A recent study among newly diagnosed type 2 diabetes in Bangladesh reported 84% of patients had average to poor basic knowledge about their disease [8].

Mobile phone interventions for health are an emerging, rapidly-evolving practice and have been used to improve delivery of health services in many countries of the world [9]. Mobile phones can be a low cost solution to provide health education and increase adherence to treatment for people with chronic diseases like diabetes [10]. A study using mobile phone SMS by nurses in Korea found that SMS for 6 months reduced HbA1C in diabetic patients to 1.15% at 3 months and 1.05% at 6 months compared with baseline in the intervention group [11]. Mobile phone SMS have been shown to be an effective tool for providing diabetes health education, clinic and appointment reminders, medication reminders and for building awareness about the disease [12-14]. However, no data on the effects of such an approach on patient outcomes in resource-limited settings are available to date.

### Research objectives

The objectives of this study are to:1) Study the effect of mobile phone SMS on medication adherence and control of newly-diagnosed type 2 diabetes in an urban area of Bangladesh; 2) Test the use of mobile phone SMS as clinic appointment reminders and for building awareness about diabetes and its complications; 3) Measure the impact of mobile phone SMS on quality of life of diabetes patients in Bangladesh and 4) Evaluate the cost effectiveness of a mobile phone SMS intervention.

The results of the study will help the policy-makers to understand the importance of creating healthcare systems that better meets the needs of people and develop prevention and management strategies for diabetes and other chronic disease using innovative mobile phone technologies at the national level which can be replicated in other developing countries as well.

### Hypothesis

Our primary hypothesis is that mobile phone SMS intervention is superior in reducing HbA1c in newly diagnosed type 2 diabetes compared to standard care. Secondary hypothesis is mobile phone SMS will increase diabetes knowledge, medication adherence and clinic attendance better in the interventions group than in the control group. The third hypothesis is participants in the mobile phone SMS group will have a better quality of life than participants in the control group. The fourth hypothesis is that mobile phone SMS will be a cost-effective measure for glycaemic control.

## Methods/Design

### Study site and population

This study will be implemented at a Bangladesh Institute of Health Sciences (BIHS) Hospital Out Patient Department (OPD) in Mirpur, Dhaka city which is an affiliate organization of Diabetic Association of Bangladesh (DAB). The total number of diabetic patients receiving services from the BIHS Hospital OPD from July 2010 to June 2011 was 90,146 with 2026 new registrations and total 693251 tests performed in the laboratory of the Department of Biochemistry. The average number of patients per day in the BIHS OPD will be sufficient to cover the total sample size of this study in a short period of time. We will limit our data collection to this single center where the participants represent urban areas of Dhaka and Bangladesh. Consecutive patients from this center who meet the eligibility criteria will be asked to participate in the study.

### Study type

**Figure 1:**
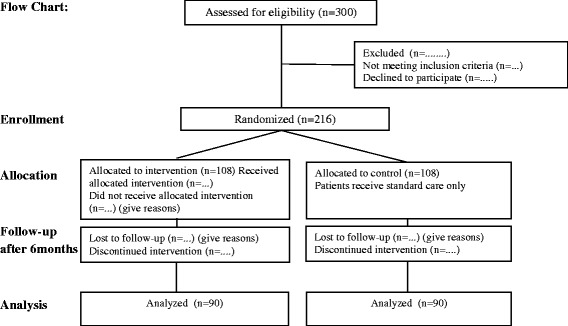
**Flow chart.** Flow chart of study participants in the trial.

We will conduct a randomized controlled trial , comparing [1] standard-of- care (n = 108) with [2] standard-of-care plus a mobile phone-based SMS intervention (n = 108) for 6 months among patients with newly diagnosed type 2 diabetes receiving treatment at the OPD of BIHS hospital in Dhaka City (Figure 1).

### Sample size

Assuming that standard care with metformin reduces HbA1c by 1% on average and the intervention adds another 0,5% [15,16]. Assuming a SD of 1, which seems reasonable and an alpha error level of 5%, the two-tailed calculation gives a power of over 90% with only 90 participants in each arm of the study. Considering 20% loss of follow up by 6 months total sample size required for this study will be 216.

**Inclusion criteria:**

- Diagnosed as type 2 diabetic patient according to WHO criteria
- Currently on oral Metformin monotherapy and able to afford cost for 6 months treatment
- Patients with a personal mobile phone
- Patients who know how to retrieve/read SMS on mobile phone (Bangla with English alphabets)
- Willingness to receive SMS for 6 months at least one SMS per day.
- Patient registered with the BIHS health centre and lives in Dhaka City.
- Provide written informed consent and agree for a follow up visit after 6 months. (SMS reminders will be sent for the follow up visit).

**Exclusion criteria:**

- Patients with other types of diabetes, such as diagnosed as type 1 diabetes, gestational diabetes.
- Patients whose clinical condition might have interfered with the study such as mental illness, other serious illness or co-morbidities requiring hospitalization.
- Patients unable to give their informed consent or agree for follow up visit.
- Patients who do not own a mobile phone or cannot read SMS messages.
- Patients who reside outside Dhaka city.

### Randomization

All the study participants will be randomized to receive either the standard care or mobile phone SMS interventions plus standard care. A central computer generated randomization list with patient ID numbers will be generated. Data collection and laboratory assays will be done by investigators masked to intervention allocation; however, study participants cannot be masked as they will receive the SMS intervention (Figure 1).

### Data collection and intervention

Data will be collected at baseline (0 month) and after 6 months through questionnaires, physical measurements and blood tests (Figure 1). We will send out mobile phone SMS to patients in the intervention group for medication reminders and health education about diabetes, its complications, diet and physical activity. SMS content has been developed using different national and international guidelines for diabetes and will be field tested for clarity and contents. The patients will be encouraged to send messages/call the study team for any queries encountered in response to the text messages. The study team will then call patients who had a problem within 2 working days. Study staff will maintain a study register to record all SMS responses and other mobile phone communications with patients in a study log.

### SMS development and testing

The SMS messages were developed by a team comprising of general physicians, an endocrinologist, an epidemiologist, a nurse and a undergraduate student. After developing the SMS messages in Bengali, we sent these messages to several individuals in the pre-test stage including persons who can only read SMS and have no formal education qualifications. After receiving the feedback, we tried to make the contents of the SMS suitable for the general population. We will ensure that all our participants in the study can read the SMS messages by themselves or someone in the family who can describe the messages to them if they do not understand. At the end of the follow-up we will evaluate if they failed to understand any of the SMS.

*Most of the 90 SMS were developed based on the principles of behavioural learning theory and transtheoretical model of behavioural change* [17-19]. *All participants in the SMS intervention group will receive the 90 SMS randomly, once a day over the 6 months period. Some examples of the SMS are provided below:*

- Diabetes is a serious disease, but it can be easily controlled
- Check your blood pressure at least for 2 times in a year and write it down
- Regular physical exercise or walking habit will help to bring your blood glucose to normal level
- Don't forget to take your medication as advised by your physician
- A planned diet and weight control will help to reduce the risk of heart disease and stroke
- Do you have any appointment with your doctor in this month? Then do not miss it.
- Have you done your physical activity, walking for 30 minutes or exercise today? If not then do it when you become free
- If you are stressed, stop your work. Go for a walk and relax!
- A healthy diet will keep you healthy and happy
- Did you take all your medication today as advised by your physician?
- Visiting your doctor timely is essential for you
- Discuss with your doctor about your concerns and worries about your condition
- Awareness about your disease and following your doctor's advice can make your life healthy and happy
- Taking medication timely will help to keep your blood sugar in control and avoid complications
- Diabetic people with disciplined life enjoys a healthy longer life.

We will attempt to replicate interview and physical measurements in a random sub-set (~5%) of the sample to evaluate repeatability. For repeatability studies, we will have about 40 participants with duplicate measures which will be sufficient to estimate kappa coefficients of agreement of inter and intra-observer reliability with a high degree of precision even for categorical variables.

### Quality monitoring and assurance

Data collection forms will be checked on a daily basis by a Research Fellow to ensure complete and accurate data entry by field staff. Cross checking of data collection forms will be performed in the field by the data collectors for inconsistency and missing data. The Principal Investigator (PI) will carry out periodic data checks and look for systematic patterns, errors, scheduling problems or incomplete forms. Every week when the team meets, the PI will pick 2 questionnaires filled in the current week by one interviewer. The PI will go through the questionnaire, question by question with the entire team, to identify incomplete/missing entries and any errors as to rectify and clear any doubts by the interviewers.

### Primary outcome measures

[Time Frame: 6 months]

- Disease progression (HbA1c level).

The primary analysis will be intention to treat which will provide relative unbiased comparisons among the intervention and control groups. It will also reduce the effects of crossover and dropout, which may break the random assignment to the treatment groups in a study.

### Secondary outcome measures

[Time Frame: 6 months]

- Diabetes knowledge (questionnaire)
- Self-reported medication adherence (Questionnaires)
- Clinic attendance (Clinic registers and questionnaires)
- Quality of Life (Self-reported questionnaires)
- Cost effectiveness of SMS interventions in reducing HbA1c

Description of outcome variables:

- Socio-demographic and economic information: Data on participant’s age, sex, religion, education, marital status, occupation, income, family size, family income, objects owned
- Mobile phone use and habits: Owns mobile phone, years of using mobile phone, can read SMS, can send SMS, frequency of sending SMS, monthly expenses for mobile phone and SMS, willingness to pay for SMS health advice/ medication reminder, maximum amount for mHealth
- Knowledge of prevention, management and complications of diabetes: Levels of knowledge and perceptions of diabetics, Risk Factors for diabetes, knowledge about prevention, management and complications of diabetes.
- Self-perceived Quality of Life Assessment: We will use EQ-5D tool and other modified tools to assess the Quality of Life for all participants. This questionnaire has been tested and validated to capture the difference in quality of life in chronic diseases patients.
- Self-reported diseases or disease prevalence: Self -reported information on different diseases and physical disability by the study participants.
- Medical History: Any known history of diabetics, CVD, hypertension, cancer, hypercholesterolemia, stroke, kidney diseases, other vascular disease, major surgery.
- Family history of diseases: Family history of diabetes, hypertension, heart diseases, stroke, kidney diseases, cancer
- Medication history: List of current and past medication taken by the patient, medication for diabetes, average monthly expenditure for medication, transportation to health facility/physician and consultation.
- Medication adherence: We will data on medication adherence of participants using different standardized tools and modifying to local context.
- Health seeking behaviour: Data will be collected on type of doctor visited during the past year, number of visits, distance travelled for consultation and expenditure for health.
- Tobacco Use (WHO modified questionnaire): A modified WHO questionnaire on use of different forms of tobacco currently and in the past with expenditure will be recorded.
- Physical Activity (GPAQ): WHO modified GPAQ questionnaire which has been validated in the fields will be used to capture information on different forms of physical activity
- Diet (WHO IMS/ Food Frequency Questionnaire): A modified and validated food frequency questionnaire will be administrated to ascertain food consumption pattern and dietary choices, type and frequency of consumption of different foods.
- Mental health status (PHQ9): The Patient Health Questionnaire PHQ 9 which is a standard tool for collecting mental health status in primary health care setting will be used.
- Life events and Disability: Life events related questions like new job, marriage, separation, injury, accident will be asked, which will be based on validated questionnaire.
- Anthropometric Measurements: Weight, Height, Mid-Upper Arm Circumference (MUAC), Waist and Hip circumference, Blood Pressure (BP), Pulse rate.
- Biological tests: Blood tests for HBA1C will be performed.

A standard operating procedure (SOP) will be developed for questionnaire administration, physical measurement, SMS, biological sample collection, transport and storage. Medical history and family history will be obtained through a face-to-face interview using structured questionnaire. The medication use will be recorded from patient's medication book that is maintained by all registered patients. The duration of diabetes, duration of hypertension and complications will be collected from patient medical records maintained by BIHS physician, review of the patient’s file and investigations.

### Data analysis

Data coding, quality control and data entry will be done following established procedures at the International Center for Diarrhoeal Diseases Research, Bangladesh (icddr, b). Precoded questionnaire will be used to minimize data coding errors. All data forms and questionnaires will be checked for errors and necessary corrections will be made before data entry. A data entry programme with built-in range and consistency checks will be used. Frequency distributions will be run to identify outliers. In all analyses, potential confounding variables and effect modifiers will be considered.

Basic presentations of data will include number and percentages of cases with diabetes by age, sex, location of residence, and in relation to different known risk factors. Chi-square tests will be used to assess the association between diabetes and risk factors. Prevalence odd ratios will be calculated for diabetes adjusted for major confounding variables (e.g. age, sex, smoking habits, socioeconomic status, nutritional status, family history of diabetes). Interaction between age and risk factors, obesity and diabetes risk factors, and nutritional status and risk factor (hypertension and diabetes risk factors) will also be examined in the logistic regression model.

Effect of intensive mobile phone SMS intervention will be examined by comparing the longitudinal change in glycated hemoglobin (HbA1c) level changes between intervention and control groups. The change in HbA1C will be defined as the difference between mean HbA1c at the first survey and minus that at the second survey and standardized for the time between surveys. We will analyze the primary outcomes with the t-test. The primary analysis will be intention to treat which will provide relative unbiased comparisons among the intervention and control groups. It will also reduce the effects of crossover and dropout, which may break the random assignment to the treatment groups in a study. Relative risk (RR) will be reported for non-adherence, with an RR less than 1 suggesting better outcome for the intervention group. As a measure of absolute effect size, we will also calculate the number needed to treat (NNT) and its associated 95% CI for both unadjusted primary outcomes. Adjusted analyses by fitting a logistic regression model to the primary outcomes with adjustments for sex, age, baseline HbA1c level will be performed.

Secondary outcomes will be compared with the χ2 test for categorical outcome. Fisher's exact test for outcomes will be used to estimate p values. Logistic regression will be used for Quality of Life (QoL) and other continuous variables. In the subgroup analysis in the intention-to-treat population, we will compare the intervention groups within each subgroup of patients with the χ2 test for the categorical outcome and logistic regression for QoL. For all models, the results are expressed as an estimate of effect size, with 95% CIs and p values. The response type throughout the study period will be analysed by categorising time since random allocation to the 6 months and doing a χ2 test between time and response type. All statistical analyses will be done using SPSS version 20 (IBM Corporation, USA).

### Health economic analysis

Primary analyses: We will measure the economic impact of the mobile phone based SMS interventions. All relevant costs incurred during the observation period will be assessed and compared between the two study arms. Cost for the intervention excluding the costs caused by the study will be considered. To calculate the economic cost of intervention we will use the CostIt (Costing Interventions templates) tool developed by WHO. The CostIt tool provides a set of separate templates for the reporting and analysis of costs at the programme, hospital, primary health facility and household levels. The following steps will be used:

1. Define the costs (identify all direct and indirect costs)
2. Evaluate the total cost of SMS intervention

The data collection tools have been designed to capture cost measurement for medication, transportation, consultation, blood tests, days lost due to illness, hospitalization costs and other associated direct and indirect costs. Costs associated with mobile phone SMS interventions will be recorded in a separate register and used for data analysis in measuring the costs which includes costs for personnel, materials and supplies, operating costs, maintenance costs, utilities, capital costs and other indirect costs. Cost for standard of care, costs for the SMS intervention and accessing the SMS intervention will be calculated. Also, costs associated with productivity gained due to improvement of health conditions will be assumed.

In order to estimate the impact of mobile phone SMS on diabetes control, we will conduct a within-trial cost-effectiveness analysis which examines cost associated with health gains. The measure of effect will be units of HbA1C reduction. In addition, a cost effectiveness-analysis will be conducted where health gains are expressed in terms of health-related quality of life as measured by the EQ-5D questionnaire. The within-trial cost effectiveness analysis will be performed with support Prof. Rolf Holle and his team at the Institute of Health Economics and Health Care Management of the Helmholtz Zentrum Muenchen, Germany.

### Ethics

All participants will be explained about the objectives, importance, risks and benefits of the research before recruitment. Participation will be completely voluntary and an appropriate written informed consent will be obtained from all participants. All participants will be given a copy of the consent form and contact details of the study coordinator for their future questions and concerns. All participants will have access to send SMS or call the study team for advice and suggestions during the study period as a compensation for participating in the study.

All participants will be assigned a unique alpha-numeric subject ID. All data-set, biological samples and clinical investigations performed for the study will be identified by the unique subject ID given to each participant. Staff at the laboratory will not have access to the confidential codes linking the subject IDs to the personal identifier. The hard-copies of all the data collection forms will be stored and locked at icddr, b and access to these files will only be given to the PI’s, and authorized staff. All computer databases will be protected by firewalls and a password-entry system. Only PI and designated staff will have access to database and files matching personal identifiers to subject IDs for the purposes of contacting study participants for follow-up. Publications and scientific presentations of the findings from the study will be presented in aggregates and identity of individual participants will be kept confidential.

Local health authority in the study site will be informed of the study and discussions will be held about management of any clinical problems arising during the study. Any participant who is discovered to have a chronic disease or risk factor(s) that require management will be appropriately informed about the condition and its implications by a medical member of the study team. They will then be referred to an appropriate service for further management.

This study has been approved by icddr, b Research Review Committee (PR# 13018) on 15 April, 2013 and Ethical Review Committee on 28^th^ May 2013.

## Discussion

This article describes the protocol for a randomized control study to measure the effects of mobile phone SMS on diabetes knowledge, medication adherence, clinic attendance and glycemic control for patients with type 2 diabetes in an urban area of Bangladesh. This study is the first in developing countries, to the best of our knowledge, to measure the impacts of mobile phone SMS for diabetes. One of the most significant barriers to effective treatment of type 2 diabetes is a lack of awareness and education about diabetes, its complications and optimal way to treatment which can be addressed by mobile phone interventions for knowledge generation, awareness and behaviour change communications.

Although a nationally representative and multi-center, cluster randomized controlled trial is optimum from the methodological perspective for this study, due to lack of resources and time constraints we have to limit our study to a single centre. However, data gathered from this study will allow us to evaluate the effectiveness and cost-effectiveness of SMS interventions in reducing HbA1c from within the trial. In recent years there are numerous attempts to integrate mobile phones for health interventions and this study can direct future research in Bangladesh for diabetes management in clinical settings using innovative mobile technologies.

Participants will be identified before randomization in an attempt to avoid bias. We will initially inform the participants about the study and after obtaining informed consent will conduct the interviews and obtain blood samples for HbA1c. We will then randomize the participants using a random allocation software to the intervention and control arms.

Using SMS as tools for medication and appointment reminders; disseminate health information and life-style messages are easy technology that can be applied by persons with minimum technical knowledge and skills. In this study, we will send the SMS to participants using a internet modem with build-in software for sending SMS and bulk messages. As the SMS will be written in Bengali using English alphabets we assume it will not be very difficult for participants to read and understand. However, as the literacy rate of Bangladesh has been reported to be only 62.5%, the ability to read the SMS messages were considered in the inclusion criteria which does not automatically translate into the ability to understand the contents of the SMS.

Outcomes between the two groups will be measured after 6 months by standardised questionnaires, review of medical records and patient medication book, physical measurements and biological sample to determine effects of the intervention on outcomes. Although the short time period will not allow us to evaluate the impact of mobile phone SMS on development of diabetic complications, this will provide us with a baseline data which can be tested on future extension of this study. Furthermore, we expect that the SMS will have some impact on lifestyle outcome measures than on metabolic parameters because a lifestyle change must occur before we can measure an effect on metabolic parameters. We expect the metabolic parameters to have stronger long-term effects. Also, it is expected that the SMS intervention will possibly help to increase knowledge about diabetes and its complications, leading to better medication adherence which ultimately might lead to better glycaemic control.

Although we originally intended to perform detailed health economic analysis for this study we considered that secondary analyses will be performed depending on positive results of the primary analyses and the availability of human resources. In the secondary analyses, we will attempt to combine the short-term evidence from the SMS intervention study with expected long-term effects and outcomes for the patients. These analyses will use decision-analytic models such as Markov or state transition models to describe the disease process.

Each unit of HbA1c reduction will be converted to a health outcome unit for example DALY (Disability Adjusted Life Years) gained and compared between non-intervention and intervention in terms of financial unit. The DALY extends the concept of potential years of life lost due to premature death (PYLL) to include equivalent years of “healthy” life lost by virtue of being in states other than good health. DALYs lost due to diabetes will be calculated as the sum of the years of life lost due to premature mortality (YLL) in the population and the equivalent “healthy” years lost due to non-fatal health conditions (YLD) : DALY = YLL + YLD.

When measuring the burden of diabetes, or the decrement in health due to diabetes, each year of life lost is given a weight of 1. Years lived, but in states less than full health, are given a weight between 0 and 1, with 0 representing full health. Using standardized population models such as WHO developed PopMod [20], we will calculate the benefits of the SMS intervention compared to standard of care. The outcome will be measured in terms of costs and effects. For both the standard of care and SMS intervention we will measure the expected costs and expected outcomes and the results expressed as ratio of difference in expected costs and effects.

SMS for health promotion have been well-accepted by beneficiaries and may be an effective tool for providing diabetes health education, clinic and appointment reminders, medication reminders and building awareness about the disease [12-14]. A recent study in Netherlands showed that SMS reminders improved adherence of type 2 diabetes patients, especially the precision with which the patients followed their prescribed regimen and that it was well accepted by the patients [21]. Numerous issues must be considered when designing and implementing client-centered programs, including mobile phone access, sharing of phones, language and literacy, privacy, and technological challenges. More information is needed about best practices for developing content for text message delivery and the optimal timing of messages [22].

## Abbreviations

SMS (Short Message Service): SMS is a way to send short messages using an alphabet, numbers, and symbols. SMS messages are digital information that can be transmitted over mobile networks, without Internet signal.
mHealt (Mobile Health): “the use of mobile computing and communication technologies in health care and public health” (Free et al. 2010 [9], p. 1).
Diabetes: Diabetes can be diagnosed on any of the following World Health Organization (WHO) criteria: Fasting plasma glucose (FPG) ≥7.0 mmol/l (126 mg/dl) or, 75 g oral glucose tolerance test (OGTT) with FPG ≥7.0 mmol/l (126 mg/dl) and/or 2 hour plasma glucose ≥11.1 mmol/l (200 mg/dl) or, Glycated haemoglobin (HbA1c) ≥6.5% / 48 mmol/l, or Random plasma glucose ≥11.1 mmol/l (200 mg/dl) in the presence of classical diabetes symptoms.
Asymptomatic individuals with a single abnormal test should have the test repeated to confirm the diagnosis unless the result is unequivocally elevated. Where a random plasma glucose level ≥5.6 mmol/l (≥100 mg/dl) and <11.1 mmol/l (<200 mg/dl) is detected: a FPG should be measured, or an OGTT performed, or an HbA1c measured.
Compliance: Has been defined as “the extent to which a person’s behavior coincides with medical advice”. Noncompliance then essentially means that patients disobey the advice of their health care providers. Patient noncompliance is attributed to personal qualities of the patients, such as forgetfulness, lack of will power or discipline, or low level of education.
Adherence: Adherence is defined as the “active, voluntary, and collaborative involvement of the patient in a mutually acceptable course of behavior to produce a therapeutic result.”
HbA1C: HbA1c indicates the level of diabetes control over the last 2-3 months and it is directly related to the occurrence of complicating events later in life
